# High yield purification of full-length functional hERG K^+^ channels produced in Saccharomyces cerevisiae

**DOI:** 10.1186/s12934-015-0193-9

**Published:** 2015-02-07

**Authors:** Karen Molbaek, Peter Scharff-Poulsen, Claus Helix-Nielsen, Dan A Klaerke, Per Amstrup Pedersen

**Affiliations:** Department of Veterinary and Clinical Animal Science, University of Copenhagen, Dyrlaegevej 100, Frederiksberg, DK-1870 Denmark; Department of Biology, University of Copenhagen, Universitetsparken 13, Copenhagen OE, DK- 2100 Denmark; Department of Environmental Engineering, Technical University of Denmark, Miljoevej building 113, Kgs Lyngby, 24105 Denmark; Aquaporin A/S, Ole Maaloesvej 3, Copenhagen N, DK-2200 Denmark; Laboratory for Water Biophysics and Membrane Technology, University of Maribor, Smetanova ulica 17, Maribor, SL-2000 Slovenia

**Keywords:** hERG, Potassium channel, Membrane protein production and purification, Functional expression, Yeast, Cardiac action potential, Drug screening, Long QT, Torsades de Pointes

## Abstract

The hERG potassium channel is essential for repolarization of the cardiac action potential. Due to this vital function, absence of unintended and potentially life-threatening interactions with hERG is required for approval of new drugs. The structure of hERG is therefore one of the most sought-after. To provide purified hERG for structural studies and new hERG biomimetic platforms for detection of undesirable interactions, we have developed a hERG expression platform generating unprecedented amounts of purified and functional hERG channels. Full-length hERG, with or without a C-terminally fused green fluorescent protein (GFP) His _8_-tag was produced from a codon-optimized hERG cDNA in *Saccharomyces cerevisiae*. Both constructs complemented the high potassium requirement of a knock-out *Saccharomyces cerevisiae* strain, indicating correct tetramer assembly *in vivo*. Functionality was further demonstrated by Astemizole binding to membrane embedded hERG-GFP-His _8_ with a stoichiometry corresponding to tetramer assembly. The 156 kDa hERG-GFP protein accumulated to a membrane density of 1.6%. Fluorescence size exclusion chromatography of hERG-GFP-His _8_ solubilized in Fos-Choline-12 supplemented with cholesteryl-hemisuccinate and Astemizole resulted in a monodisperse elution profile demonstrating a high quality of the hERG channels. hERG-GFP-His _8_ purified by Ni-affinity chromatography maintained the ability to bind Astemizole with the correct stoichiometry indicating that the native, tetrameric structure was preserved. To our knowledge this is the first reported high-yield production and purification of full length, tetrameric and functional hERG. This significant breakthrough will be paramount in obtaining hERG crystal structures, and in establishment of new high-throughput hERG drug safety screening assays.

## Background

The lack of high resolution structures is a common theme among membrane proteins. In contrast to the more than 90,000 structures known for water soluble proteins [1], only 512 membrane protein structures have been determined so far [2] (December 2014). This bias is also reflected by the fact that membrane proteins constitute around 30% of all proteins [3], and that 60% of all known drugs target a membrane protein. Similarly, membrane proteins are the most prominent targets for new drugs [4]. Thus, the need to increase our understanding of membrane proteins is crucial. Potassium channels constitute a particularly interesting family of membrane proteins as they are very important targets for various neuropathologies [5] and heart conditions [6] as reviewed recently in Tian et al. 2014 [7]. Consequently, there is a great interest in solving the structures of these and other ion channels. However, such studies are impaired by difficulties in recombinant production of large amounts of functional channels, and establishment of optimal conditions for purification of stable and functional protein. Thus far, high resolution structures of fourteen different K ^+^ channels are available, out of which seven are of archaic and bacterial origin, seven mammalian and of these only three are human [2] (December 2014).

The human *Ether-à-go-go* related gene hERG encodes the pore forming *α*-subunit of a voltage gated potassium channel [8]. The hERG channel is most abundantly expressed in the heart where the channel is involved in repolarization of the cardiac action potential, by conducting the rapid component of the delayed rectifier potassium current, IKr [9]. HERG is also expressed in brain [10], intestine [11,12] and in the endocrine system [13]. The hERG channel has been the focus of much attention due to the identification of hERG mutations [14] that cause severe heart conditions such as long QT syndrome and Torsades de Pointes. Similarly, the hERG channel has been shown to interact with a variety of structurally diverse drugs, some of which caused fatal arrhythmias, and have been withdrawn from the market [15]. Consequently it has become a requirement by the U.S. food and drug administration (FDA) and the European Medicines Agency (EMEA) to analyze the activity of hERG in presence of potential new drugs [16,17].

**Figure 1:**
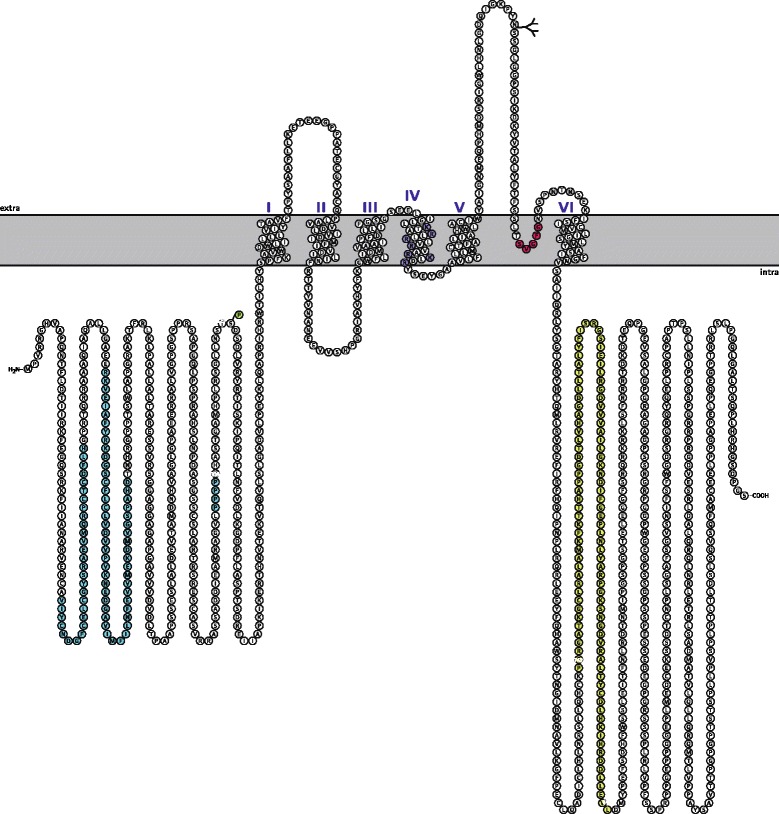
**Structural model of the 1159 amino acids long hERG** ***α*** **-subunit.** The figure is based on data from http://www.uniprot.org/uniprot/Q12809 and plotted into textopo. The light gray area visualizes the lipid membrane. The 400 amino acids long N-terminal contains a PAS domain (residues V41-H70), a PAC domain (residues R92-D144), a compositional bias poly prolin stretch (residues P297-P300) all in sky blue and a phosphorylation site at residue S320 (green). The channel part of the protein consists of the six transmembrane domains (S1-S6). The charged residues in segment 4 responsible for voltage sensing (residues K525,R528,R531,R534,R537,K538 and R541) are marked in light blue. A predicted glycosylation site at residue N598 is labeled with a branch. The canonical SVGFG signature motif of the selectivity filter (residues S624- G628) located in the loop between segment 5 and 6 is shown in wild strawberry. The intramembrane region of the protein may actually span from residue 612-632, but here only residues S621-N629 are shown residing within the membrane. The C-terminal cyclic nucleotide binding like domain (cNBD) is marked in spring green and spans residues P742 - L842.

The fully assembled channel, termed Kv.11.1., is a homotetrameric complex of the 1159 amino acids hERG *α*-subunit [8]. Figure 1 illustrates that each subunit has six transmembrane segments (TM1 through TM6), a 403 amino acids N-terminal and a 500 amino acids C-terminal. The pore loop between TM5 and TM6 [18] carries the canonical K ^+^ channel sequence SVGFG, that along with TM5 and TM6 comprise the K ^+^ selective pore of the protein [1]. The four charged arginine residues mainly responsible for voltage sensing are located in TM4 [19]. The cytosolic N- and C-terminal domains are believed to encompass a Per-Arnt-Sim (PAS) domain [20] and a cyclic nucleotide binding domain cNBD, respectively [8].

Although the great importance of hERG in heart physiology and drug development has long been recognized, the three dimensional atomic resolution structure of the entire 1159 amino acid channel protein has not yet been determined. Thus, structural insight into the molecular mechanism of hERG function has been restricted to homology models based on crystal structures of similar voltage gated K ^+^ channels such as the significantly shorter (282 amino acids) KvAP channel from the archae *Aeropyrum pernix* [21] and the 499 amino acids Kv1.2 K ^+^ channel from rat [22]. However, the N-terminal 135 residues of hERG has been crystallized revealing that both gating and subunit assembly are associated with this part of the protein [20]. Consequently, characterization of hERG has been restricted to electrophysiology [23], flux measurements [24] and ligand binding [25].

Protein chemical and structural studies of hERG have been hampered by lack of expression systems that can provide large quantities of functional hERG protein in its active tetrameric form. Purification of recombinant, full-length hERG from Sf9 insect cells resulted in monomeric hERG subunits, which seemed to be correctly folded but non-functional [26]. A protein fusion approach that stabilizes the tetrameric structure of hERG was recently presented [27]. In this study, a heavily engineered channel in which important parts of the channel were replaced with a dimerization domain from the yeast Gcn4 transcription factor was produced in HEK cells and shown to maintain its tetrameric structure during purification. Another approach involved replacing the transmembrane segments of Kv1.2 with TM1-TM6 from hERG and expression in *Pichia pastoris* [28]. However, the hERG cytosolic N- and C-termini were absent, the expressed S1-S6 chimaeras were partly glycosylated and the purified chimaeras contained degradation products. Cell-free biosynthesis and subsequent incorporation into biomimetic membranes have also been demonstrated using the same TM1-TM6-domain [29]. The ability of the membrane embedded hERG fragments to bind known inhibitors suggests potential applications in drug screening. However, exclusion of the N- and C-terminal residues involved in the gating mechanism and subunit assembly may be a draw back. In the present study we demonstrate that substantial amounts of full length, functional and tetrameric hERG can be produced in our *S. cerevisiae* high-copy vector expression system [30-32]. We show that the recombinant hERG channel can be purified in its native, functional tetrameric form. To our knowledge this is the first successful purification of functional tetrameric hERG channels. This may facilitate crystallization and biochemical characterization of this important channel and serve as starting point for inexpensive large scale biomimetic high-throughput screening systems for identification of drug candidates free of unintended interactions with hERG.

## Results

### hERG expression plasmids

**Figure 2:**
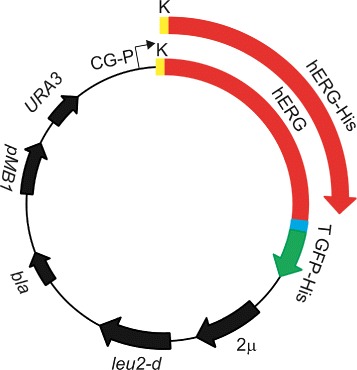
**Structural map of the hERG-TEV-GFP-His**_**8**_ **and hERG-His**_**10**_ **expression plasmids.** Abbreviations used: CG-P, a hybrid promoter carrying the GAL10 upstream activating sequence fused to the 5 ^′^ non-translated leader of the cytochrome-1 gene; T, Tobacco Etch Virus (TEV) cleavage site; GFP-His _8_, yeast enhanced GFP cDNA fused to eight histidine codons; 2 *μ*, the yeast 2 micron origin of replication; *leu2-d*, a poorly expressed allele of the *β*-isopropylmalate dehydrogenase gene; *bla*, a *β*-lactamase gene; pMB1, the pMB1 origin of replication; *URA3*, the yeast orotidine-5 ^′^-phosphate decarboxylase gene. Rapid construction of expression plasmids was carried out by *in vivo* homologous recombination in *S. cerevisiae*.

In order to maximize the yield of the hERG-TEV-GFP-His _8_ and hERG-His _10_ fusion proteins we constructed the expression plasmids outlined in Figure 2. Each fusion is expressed from a strong galactose inducible CYC-GAL promoter whose activity is further enhanced in the host strain PAP1500 through regulated overexpression of the Gal4 transcriptional activator [30]. To increase hERG protein production the vector carries the crippled *leu2-d* gene that facilitates an ultra-high plasmid copy number in the range of 200 to 400 per cell in response to leucine starvation [33]. The combined features of the PAP1500 expression system was chosen due to our previous success with this system for high yield expression of a variety of eukaryotic membrane proteins [30-32,34].

### *S. cerevisiae* produces functional membrane integrated hERG channels

**Figure 3:**
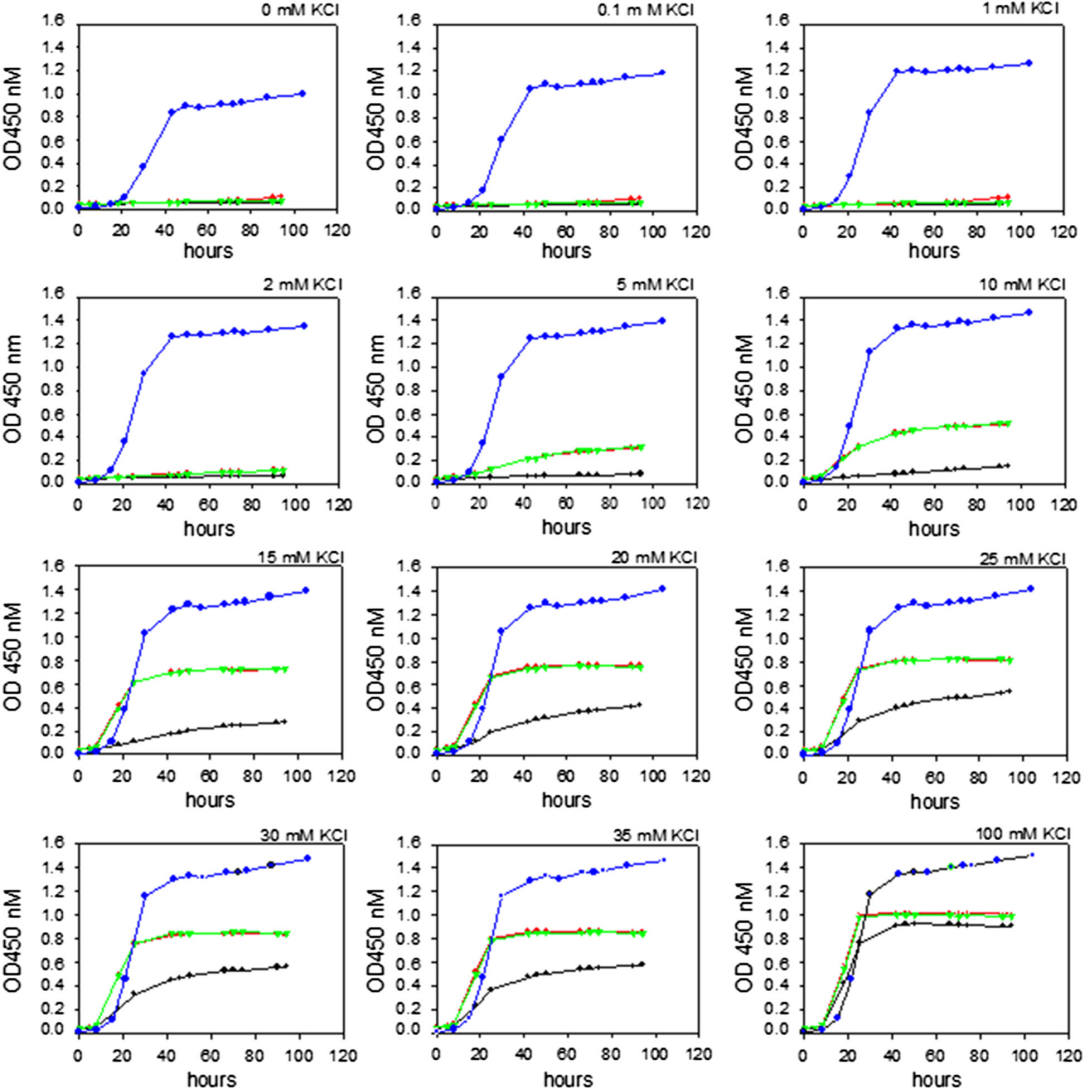
**Yeast complementation assay.** Growth in microplates at room temperature of the *trk1* *Δ*, *trk2* *Δ* yeast strain PAP7111 expressing hERG-TEV-GFP-His _8_ (green), hERG-His _10_ (red), no hERG channel (black) or the yeast wild type strain BY4741 (blue) for 96 hours in presence of the indicated KCl concentrations.

Before developing optimal expression and purification protocols we found it crucial to analyse whether *S. cerevisiae* has the capacity to assemble the homotetrameric hERG channel in a functional form in the plasma membrane and to determine if presence of the TEV-GFP-His _8_ tag interferes with hERG functionality. To address these issues we investigated the ability of the TEV-GFP-His _8_ or His _10_ tagged hERG channel to complement the potassium requirement of the *trk1Δ*, *trk2Δ* yeast strain PAP7111 at 11 different KCl concentrations. To relate the complementation capacity of the hERG channels to natural yeast endogenous potassium transport we included a wild type yeast strain in the growth assays. The growth curves in Figure 3 show that wild type yeast cells grew at extremely low potassium concentrations, even in presence of only the potassium contamination present in the chemicals used to prepare the growth medium. In contrast yeast cells producing hERG-TEV-GFP-His _8_, hERG-His _10_ protein or no hERG protein did not show any growth at KCl concentrations below 2 mM. However, yeast cells producing either of the two hERG fusions grew significantly faster at potassium limited conditions than strain PAP7111 harbouring the expression vector pEMBLyex4. Thus, PAP7111 producing the fusions grew at 5 mM and 10 mM KCl, whereas no or negligible growth was detected for PAP7111 harbouring the pEMBLyex4 expression vector. At 100 mM the three PAP7111 transformants proliferated with almost the same growth rate. This shows that the heterologously expressed hERG channels are functional and suggests that the native tetrameric structure accumulates in the yeast plasma membrane. Furthermore, since both the TEV-GFP-His _8_ and the His _10_-fusions complements the potassium transport defect of PAP7111 equally well, we conclude that the TEV-GFP-His _8_ tag does not influence channel activity or membrane targeting to any detectable extent.

### A high membrane density of hERG-TEV-GFP-His_**8**_ is obtained at 15°C

**Figure 4:**
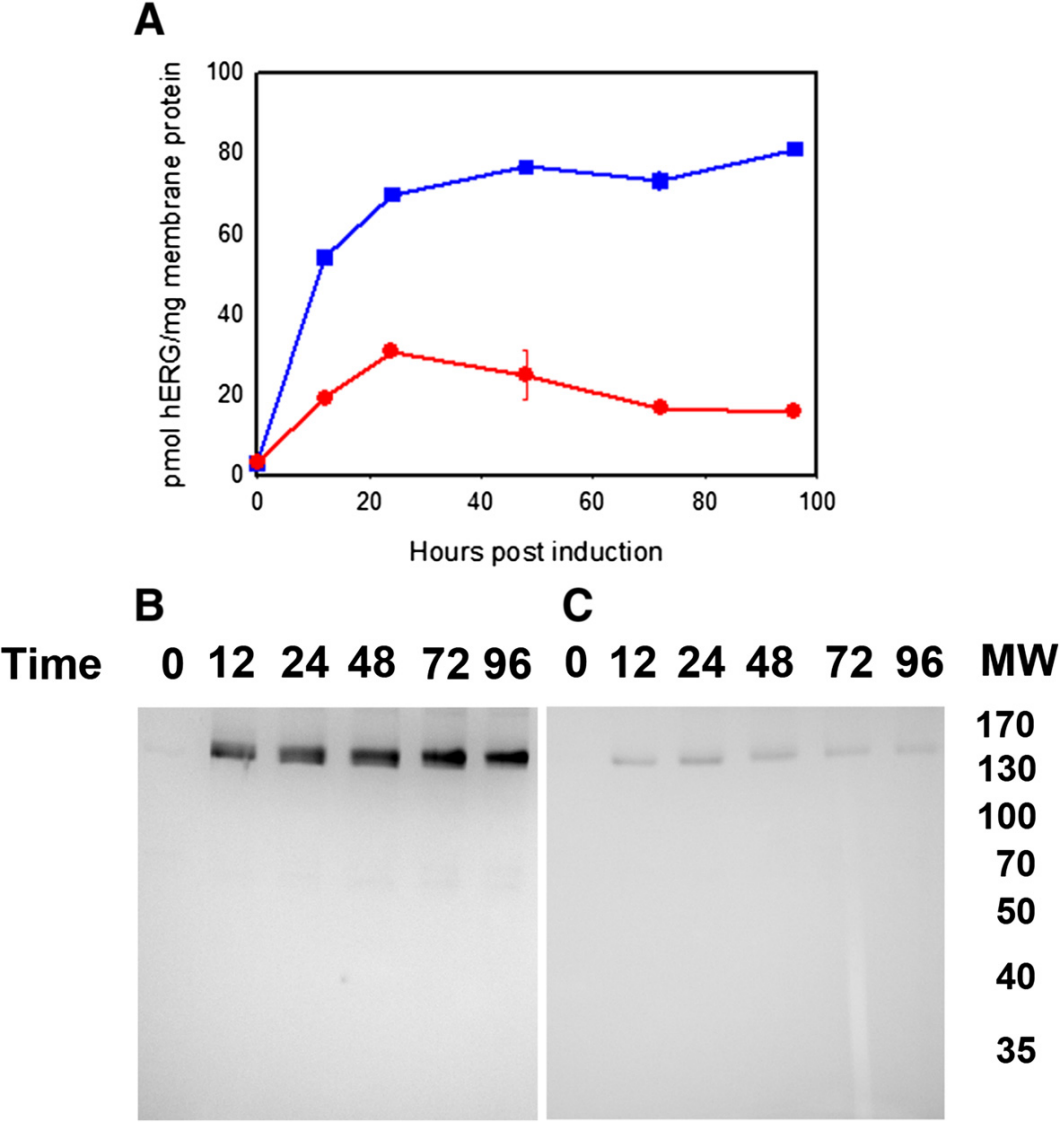
**Time and temperature dependent accumulation of hERG-TEV-GFP-His**_**8**_ **.** **A)** Exponentially growing cells cultivated at room temperature in expression medium until OD _450_ = 1.0 were separated in two. One half was transferred to 15°C while the other half was inoculated at 30°C. After 15 minutes of thermo-equilibration, production of hERG-TEV-GFP-His _8_ was induced by addition of Galactose (T = 0). Fluorescence was determined in duplicates of crude membranes isolated from yeast cells induced for the indicated periods of time at either 15°C (blue line squares) or 30°C (red line circles). Fluorescence was translated into pmol hERG protein/mg total membrane protein using a GFP standard curve. Standard deviations of duplicates are shown as error bars. **B)** In-gel fluorescence of 80 *μ*g crude membranes prepared from the cultures induced at 15°C used in figure A. **C)** In-gel fluorescence of 80 *μ*g crude membranes prepared from the cultures induced at 30°C used in figure A. Lanes are marked with time of hours post induction.

In order to maximize production of recombinant hERG we used our production strain PAP1500 [30] and identified the expression conditions giving the highest hERG-TEV-GFP-His _8_ membrane density. We therefore determined the kinetics of fluorescence accumulation in crude membranes isolated from PAP1500 cells induced for expression at 15°C or 30°C. Based on previous experience [31,32] these temperatures were selected as expression at 15°C usually improves yield and quality of recombinant membrane proteins and 30°C is the optimal temperature for yeast growth. The accumulation profiles in Figure 4 show that production at 15°C caused hERG-TEV-GFP-His _8_ to accumulate to a high membrane density that stabilized over time, whereas at 30°C fluorescence peaked after 24 hours at a much lower level and subsequently declined. Production at 15°C resulted in accumulation of 80 pmol hERG/mg protein in crude membranes, corresponding to 1.6% of the total cellular membrane protein content.

### HERG-TEV-GFP-His _8_ accumulates in the plasma membrane

**Figure 5:**
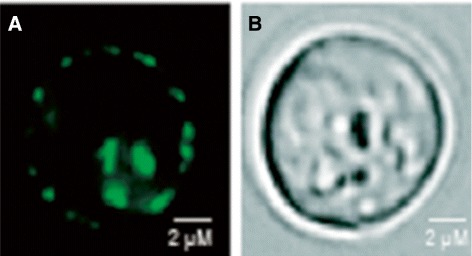
**Live cell bioimaging of PAP1500 yeast cells expressing the hERG-TEV-GFP-His**_**8**_ **fusion protein.** Yeast cells were grown in expression medium at room temperature, transferred to 15°C and induced with 2% Galactose for 24 hours. **A**, GFP fluorescence; **B**, differential interference contrast image (DIC).

In native tissue the hERG channel is located in the plasma membrane and as seen in Figure 5 live cell bioimaging revealed that recombinant hERG-TEV-GFP-His _8_ expressed in our production strain PAP1500 also localized to the yeast plasma membrane. Membrane-integrated accumulation of the hERG-TEV-GFP-His _8_ fusion also indicates that hERG was correctly folded and functional [35] in our production strain.

### HERG is N-glycosylated in *S. cerevisiae*

**Figure 6:**
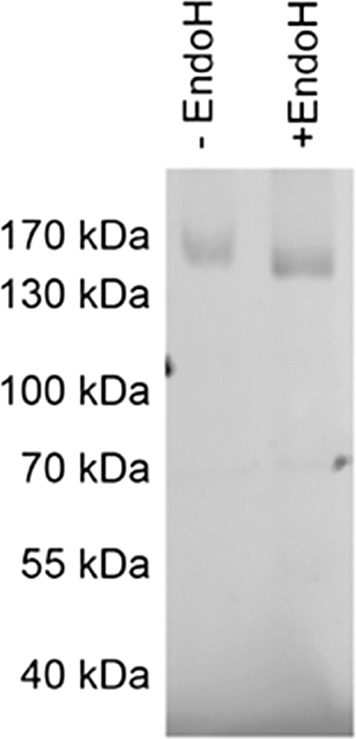
**Endo-H treatment of crude yeast membranes under non-denaturing conditions.** Crude yeast membranes (80 *μ*g) were treated with Endo-H as described in methods in a non denaturing buffer at 4°C over night. Untreated crude membranes (lane 1) or Endo-H treated membranes (lane 2) were separated by SDS-PAGE and analyzed by in-gel fluorescence. The only bands visible are the hERG-TEV-GFP-His _8_.

It has previously been shown that hERG is N-glycosylated when produced in HEK293 cells [36]. To address whether hERG-TEV-GFP-His _8_ produced in *S.cerevisiae* is N-glycosylated we treated crude membranes with Endo-glycosidase H and analyzed the digestion by SDS-PAGE and in-gel fluorescence. Data in Figure 6 show that hERG is also N-glycosylated in *S. cereviseae* as Endoglycosidase-H treatment increased the electrophoretic mobility of hERG-TEV-GFP-His _8_. The data also show that N-glycosylation can be removed completely under the non-denaturing conditions applied in Figure 6.

### Astemizole binds with high affinity to membrane embedded hERG with a single binding site per tetramer

**Figure 7:**
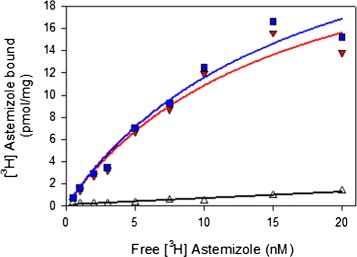
**Concentration dependent binding of [** ^**3**^ **H] Astemizole to crude membranes.** Crude membranes corresponding to 200 *μ*g total membrane protein content were incubated with [ ^3^H]-Astemizole in ranges of 0.5 - 20 nM with and without a surplus of 10 *μ*M unlabeled Astemizole at 15°C for 2 hours. Membrane bound ligand was quantified by liquid scintillation counting and normalized to sample volumes and protein content to determine total (blue squares), unspecific (open triangles) and specific binding (red triangles) as pmol bound [ ^3^H]-Astemizole/mg crude membrane protein. Nonlinear regression was used to fit the experimental data to a Michaelis-Menten equation as described in Methods.

To test the quality of membrane embedded hERG-TEV-GFP-His _8_ in PAP1500 we determined the affinity and capacity for Astemizole binding to crude membranes isolated from yeast cells induced for hERG production at 15 °C. Astemizole was selected because it is a known specific hERG ligand with high affinity and binding capacity is known to correlate with patch clamp electrophysiological measurements [37,38]. It can be seen from Figure 7 that the dissociation constant, K _*D*_, for Astemizole binding was 15 nM while the binding capacity was estimated to 28 pmol/mg crude membrane protein. The high affinity indicates that the hERG channel is correctly assembled in the yeast plasma membrane and the binding capacity of 28 pmol/mg crude membrane protein fits quite well with a single binding site per tetramer as the hERG-GFP protein density was estimated to 80 pmol/mg total protein in crude membranes.

### Fos-Choline-12 efficiently solubilizes hERG-TEV-GFP-His _8_

**Figure 8:**
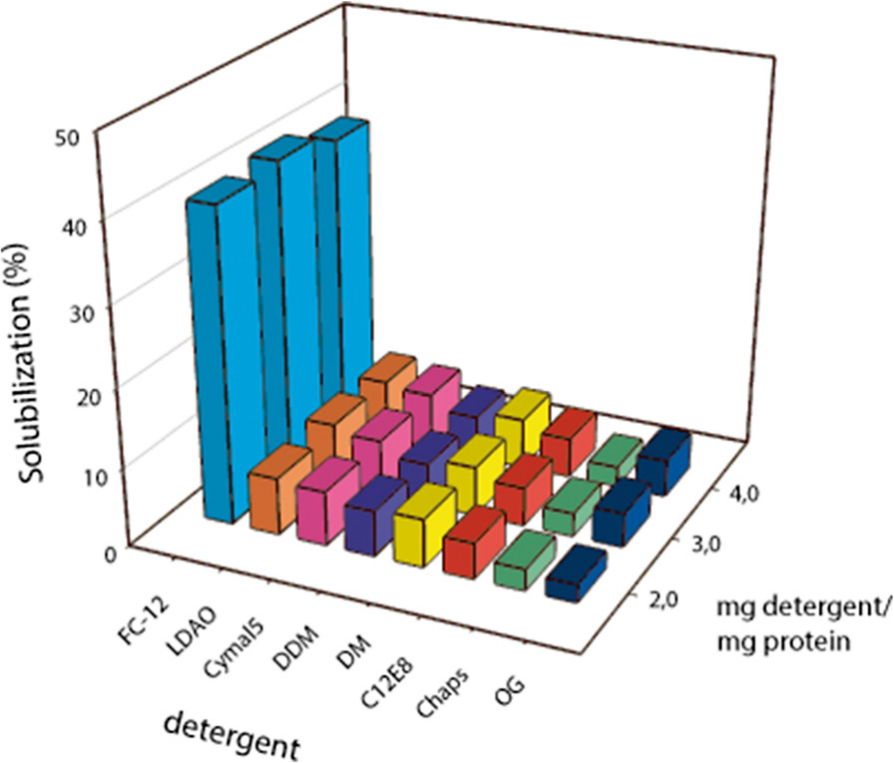
**Detergent screen of crude membranes from PAP1500 producing hERG-TEV-GFP-His**_**8**_ **at 15°C.** Membrane proteins were solubilized as described in the Methods section, at the indicated detergent/protein ratios and a cholesteryl-hemisuccinate concentration of 4.25; 2.8 or 2 mg/ml for the 3 ratios, respectively. Abbreviations; FC-12, Fos-Choline-12; LDAO lauryldimethylamine N-oxide; CYMAL5, 5-Cyclohexyl-1-pentyl- *β*-D-maltoside ; DDM, n-Dodecyl- *β*-D-maltopyranoside; DM, n-Decyl- *β*-D-maltopyranoside; C _12_ *E*_8_, Octaethylene glycol monododecyl ether; CHAPS, 3-[(3-cholamidopropyl) dimethylam-monio] -1-propanesulfonate; OG, n-Octyl- *β*-D-glucopyranoside. Solubilization was determined as GFP fluorescence of solubilized protein normalized to GFP fluorescence in the crude membranes used for solubilization.

To find a suitable detergent for solubilization of the hERG-TEV-GFP-His _8_ fusion, a solubilization screen was set up with eight different detergents. Based on previous experience [32] solubilization was carried out in a mixture of detergent and cholesteryl-hemisuccinate (CHS) to stabilize the hERG-TEV-GFP-His _8_ fusion during and after extraction from the membranes. Figure 8 shows that a protein:FC-12:CHS ratio of 1:3:1.5 (w/w/w) most efficiently solubilized the channel from crude yeast membranes, yielding approximately 40% solubilized protein. Three subsequent solubilization experiments using FC-12 and CHS gave on average 48% solubilization of hERG with a standard deviation of 9% (data not shown). The remaining detergent:CHS mixes only resulted in approximately 5% solubilization of the hERG channel.

### FSEC reveals that CHS and Astemizole improve the quality of solubilized hERG-TEV-GFP-His _8_

**Figure 9:**
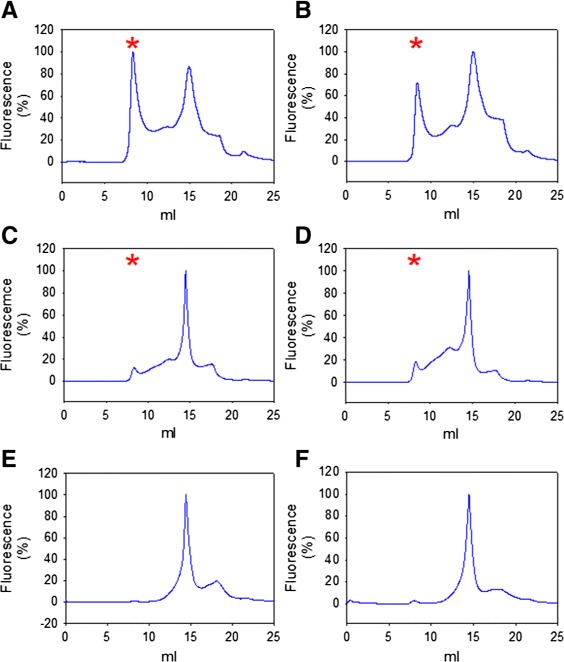
**FSEC profiles of solubilized crude membranes.** Membranes were isolated from yeast cells induced for hERG-TEV-GFP-His _8_ production at 15°C for 48 hours. Membranes were solubilized at a concentration of 2 mg/ml in FC-12 at a 3:1 detergent to protein ratio without any supplement **(A)**, supplemented with 5 mM KCl **(B)**, supplemented with 5.1 mg/ml cholesteryl-hemisuccinate **(C)** or both **(D)**, with cholesteryl-hemisuccinate and 1 *μ*M Astemizole **(E)** or with cholesteryl-hemisuccinate without Astemizole **(F)** as described in Methods section. Solubilizations **E** and **F** were separated in presence of 1 *μ*M Astemizole. Solubilized membrane proteins were separated on a Superose 6 10/300 GL column. Molecular weight markers (GE Healthcare Life Science) separated on the same column eluted as follows: Blue Dextran 2000, 2000 kDa at void volume 8 ml (marked with an asterisk), Thyroglobulin 669 kDa at 12.5 ml, Ferritin 440 kDa at 14.5 ml, Aldolase 158 kDa at 16.3 ml, Conalbumin 75 kDa at 17.3 ml, Ovalbumin 44 kDa at 17.6 ml.

To identify conditions that improve the quality of solubilized hERG-TEV-GFP-His _8_ we performed FSEC analysis on membranes solubilized in presence or absence of KCl, CHS and Astemizole. As seen from Figure 9 addition of CHS increased solubilization efficiency and resulted in an almost monodisperse elution profile with a reduced amount of aggregated protein eluting in the void volume. Presence of 5 mM KCl during solubilization did not increase protein quality irrespective of presence of CHS. Presence of 1 *μ*M Astemizole during solubilization and size exclusion chromatography or only during the chromatographic step resulted in a further improved FSEC profile showing a narrower and more symmetrical elution peak (Figure 9E and F). This demonstrates that Astemizole binds quantitatively to the solubilized hERG channel and that the solubilized channel has maintained its tetrameric structure. This is further supported by the fact that the 156 kDa hERG-TEV-GFP-His _8_ fusion eluted as an approximately 620 kDa protein according to the elution profile of the MW standards, even though the amount of detergent in the hERG detergent complex is unknown. The observation that presence of Astemizole was only required during size exclusion chromatography to improve the FSEC profile indicates that the broader FSEC profile observed in presence of only FC-12 and CHS may reflect the flexibility of the channel and not partly inactivated channels.

### Ni-affinity purification results in highly pure hERG-TEV-GFP-His _8_ protein

**Figure 10:**
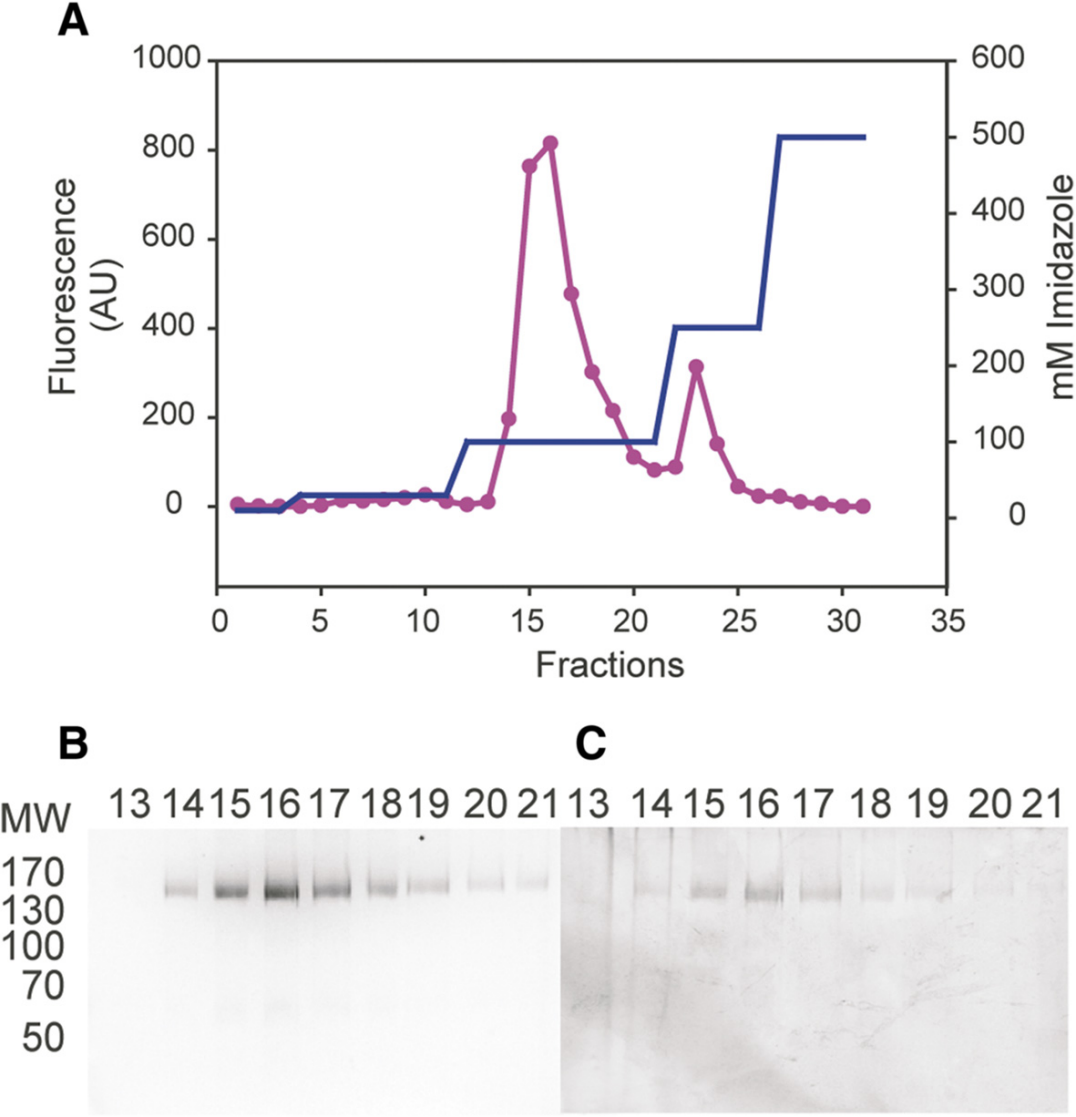
**Purification of hERG-TEV-GFP-His**_**8**_ **by Ni-affinity chromatography.** HERG-TEV-GFP-His _8_ was solubilized in FC-12/CHS and incubated with Ni-resin over night at 4°C, as described in the Methods section. The Ni-resin was poured on a column and hERG-TEV-GFP-His _8_ was eluted from the Ni-resin using the indicated imidazole step gradient (blue). Fluorescence was measured in each fraction to estimate the elution profile of the hERG-fusion (pink). **B)** In-gel fluorescence of Ni-affinity purified hERG-TEV-GFP-His _8_ separated by SDS-PAGE in an 8% gel. Lanes contain fractions 13-21 from **A**. **C)** Coomassie stain of the SDS-PAGE gel in **B**.

To purify the hERG-TEV-GFP-His _8_ fusion we solubilized crude membranes in FC-12:CHS at a protein:detergent:cholesterol ratio of 1:3:1 (w/w/w). As seen from Figure 10 the hERG-TEV-GFP-His _8_ protein eluted as a major peak at 100 mM imidazole. In-gel fluorescence and Coomassie staining of SDS-PAGE separated peak fractions revealed a high degree of purity (Figure 10B and 10C) since only full-length fluorescent protein with a molecular weight of the expected 156 kDa was visible in the Coomassie stained gel.

### Purified recombinant hERG binds Astemizole

**Figure 11:**
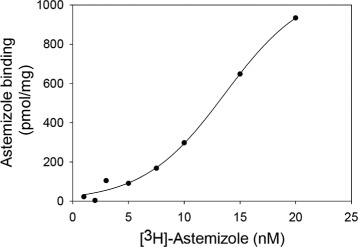
**Binding affinity and capacity of [** ^**3**^ **H]-Astemizole to purified hERG-TEV-GFP-His**_**8**_ **.** 0.1 *μ*g affinity purified hERG was incubated with [ ^3^H]-Astemizole in ranges of 0.5 - 20 nm with or without a surplus of 10 *μ*M unlabelled Astemizole at 15°C for 2 hours. [ ^3^H]-Astemizole binding was quantified by scintillation counting as described in the Methods section. All solutions contained 1.5 mg/ml FC-12 and 0.5 mg/ml CHS. The experimental data were fitted to a sigmoid curve as described in the Methods section.

To study the quality of detergent solubilized and purified hERG-TEV-GFP-His _8_ we determined the affinity and capacity for Astemizole binding. Data in Figure 11 show that the purified channel was able to bind Astemizole with an affinity of 13.8 nM and a capacity of 1.1 nmol/mg fusion protein. Since the theoretical binding capacity for the pure 156 kDa hERG-TEV-GFP-His _8_ fusion amounts to 1.4 nmol/mg fusion protein, this strongly suggests that the native tetrameric structure was preserved during detergent solubilization and purification. The discrepancy between the actual and theoretical binding capacity may be explained by the presence of protein contaminants in the affinty purified hERG-TEV-GFP-His _8_ preparation. The binding affinity for Astemizole measured for the detergent solubilized hERG-TEV-GFP-His _8_ fusion and for the membrane embedded fusion were similar; 13.8 nM and 15.0 nM respectively. In contrast to the ligand binding curve in Figure 7 based on membrane embedded hERG-TEV-GFP-His _8_, the binding curve for the purified protein did not follow a Michaelis-Menten equation but turned out to be sigmoid, which is characteristic of cooperative protein ligand interactions.

### TEV-cleavage releases the GFP-His _8_ tag from hERG

**Figure 12:**
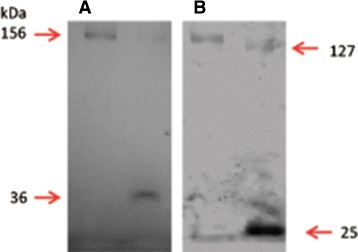
**TEV digestion of hERG-TEV-GFP-His**_**8**_ **.** Peak fraction 17 from the Ni-affinity purification in Figure 10 was dialyzed for 8 hours at 4°C and subsequently digested over night at room temperature at a hERG-TEV-GFP-His _8_: TEV ratio of 1:10 (w/w). **A)** In-gel fluorescence of undigested hERG-TEV-GFP-His _8_ protein (lane 1); TEV digested hERG-TEV-GFP-His _8_ protein (lane2). **B)** Coomassie stain of the gel in **A**. The 156 kDa band represents the hERG-TEV-GFP-His _8_ fusion, the 127 kDa band is the tag-free hERG, the 36 kDa band is the GFP-tag liberated after TEV digestion and the 25 kDa band is TEV protease.

Peak fractions from the Ni-NTA affinity purification in Figure 10 were used for TEV protease digestion at a TEV:protein ratio of 10:1 (w/w) at room temperature. Figure 12 shows the result from in-gel fluorescence (A) and Coomassie staining (B) of an SDS-PAGE separated TEV digestion. The hERG-TEV-GFP-His _8_ fusion was completely digested resulting in appearance of the fluorescent GFP-His _8_ tag as a 36 kDa protein band, whereas the full-length fusion was no longer visible (Figure 12A). A 127 kDa protein corresponding to the hERG protein released by TEV digestion was however visible in the Coomassie stained gel (Figure 12B, lane 2).

## Discussion

Potassium channels are present throughout all kingdoms and are crucial for conduction of electrical signaling [39]. Their fundamental role in cell homoeostasis makes some potassium channels obvious drug targets [40] while interfering with the activity of others may be lethal [41]. The hERG channel belongs to the latter category as inhibition of this channel can cause sudden death in otherwise healthy individuals [14]. High resolution structures of potassium channels are therefore eagerly pursued. However, their complex structure and conformational flexibility have hampered generation of high resolution 3D structures through crystallization and X-ray diffraction. Exceptions include a few voltage gated potassium channels such as the shaker channel from *Drosophila* and KvAP from the archae, *Aeropyrum pernix* [21,22]. Despite availability of milligram amounts of bacterial potassium channels, it has been challenging to obtain crystal structures of these, due to protein aggregation during purification and crystallization. The flexible voltage sensing domain is potentially the molecular cause of these challenges [21]. The only structure available for members of the family of 6-TM mammalian potassium channels to which hERG belongs is that of rat Kv1.2 [22]. Successful crystallization of rat Kv1.2 reflects that this is the only channel which has been successfully overexpressed and purified in the amount and quality required for initiating crystallization screens. Previous attempts to overexpress and purify hERG highlights these problems as purification of recombinant native, tetrameric hERG has been unsuccessful, despite investigation of a number of host organisms. Purification of recombinant hERG from Sf9 insect cells resulted in nonfunctional monomers [26]. However, the purified hERG monomers were successfully used to generate a set of monoclonal anti-hERG-antibodies [26] which may become of great value for stabilizing the tetrameric hERG protein structure during purification and crystallization as demonstrated in the KvAP channel study [21]. Direct expression into biomimetic membranes, using a cell free system has also been attempted, and proved efficient for the membrane spanning part of hERG [29] but not for the full length channel. A recent study succeeded in expressing and purifying an artificially engineered tetrameric hERG channel by introducing the dimerization domain from the Gcn4 transcription factor. However this manipulated hERG channel did not include the long N- and C-terminal parts [28].

In order to produce full length, functional and tetrameric hERG channels, we applied our yeast expression platform [30-32]. To ease quantification of recombinant hERG, determination of subcellular localization and identification of optimal solubilization and purification conditions, we produced hERG C-terminally fused to a GFP-His _8_ tag [35]. The combination of an ultra-high copy number expression vector, a yeast strain overproducing the Gal4 transcriptional activator and expression at 15°C in amino acid supplemented medium caused the hERG subunit to accumulate to a membrane density of 1.6%. This is in fact an extraordinary high membrane density as the 7TM receptors that have been successfully crystalized were purified from a membrane density of 0.2% [42]. Thus, production in our 10 L computer controlled bioreactor should generate in the vicinity of 100 milligram membrane embedded hERG channel protein (not shown).

In native tissue the hERG channel is localized to the plasma membrane. It was therefore encouraging that the recombinant hERG maintained its localization in yeast (Figure 5) as this is a good indicator of correct folding and assembly. One of the advantages of using a microbial expression host and particular *S. cerevisiae* is that an almost complete set of knock-out strains exists [43]. Availability of these strains allows application of simple complementation experiments to identify conditions that allow functional expression of many recombinant proteins. We took advantage of this by showing that both the hERG-TEV-GFP-His _8_ and the hERG-His _10_ fusion complemented the high potassium requirement of a *S. cerevisiae* strain carrying knock-outs of the *TRK1* and *TRK2* potassium transporters (Figure 3). Yeast is therefore able to assemble the hERG-TEV-GFP-His _8_ channel correctly in the plasma membrane and the GFP part does not prevent channel activity. However, as it is seen in Figure 3 an extra cellular concentration of 5 mM is required for complementation. This concentration is high compared to the extremely low potassium concentration required for growth of wild type yeast (Figure 3). In accordance with previous results [44] the wild type showed significant growth in presence of nothing but the trace ammounts of potassium inadvertently contaminating the chemicals used for preparing the growth medium. The rather high potassium concentration required for complementation by the hERG channel probably reflects that the membrane potential of around -200 mV for a wild type yeast strain is further hyperpolarized in the *trk1Δ*, *trk2Δ* yeast strain [45].

To ensure high yield, our high protein accumulation production strain PAP1500 was used for expression of hERG-TEV-GFP-His _8_. HERG produced in yeast turned out to be N-glycosylated as shown previously for HEK293 produced hERG protein [36]. We were however able to remove the glycosylation under non-denaturing conditions which is important in relation to crystallization. To ascertain that the hERG-channels produced in PAP1500 were functional too, we combined equilibrium binding to Astemizole [37] with the advantages of the GFP tag. Equilibrium binding to [ ^3^H]-Astemizole made it possible to quantify the density of correctly folded, tetrameric hERG channels, while presence of the C-terminal GFP allowed us to quantify the density of hERG-TEV-GFP-His _8_ fusion-proteins in crude membranes. The observation that the Astemizole binding capacity approached one per four hERG-TEV-GFP-His _8_ protein chains is a strong indication that the great majority of the accumulated channels is correctly folded and therefore also functional in the expression strain PAP1500, because the capacity for Astemizole binding has been shown to correlate well with patch clamp electrophysiological measurements [38]. In conclusion, our yeast expression platform assembles functional hERG channels in the plasma membrane in a quantity and quality suitable for large scale production and purification.

The next hurdle for successful purification of hERG is to identify solubilization conditions that maintain the functional, tetrameric structure. Based on our experience with the expression platform [32] we performed a solubilization screen using a mixture of detergent and cholesteryl-hemi-succinate. In agreement with a previously described solubilization screen including more than 70 detergents [26] we found that FC-12 was the only detergent that solubilized the recombinant hERG channel at an acceptable level. Although some proteins have been crystallized in FC-12 [46] it may however not be the most appreciated detergent for crystallization as it is generally regarded a quite harsh detergent [47]. However, our FSEC analysis showed that a very sub-optimal elution profile indicative of aggregated protein could be rescued by solubilization in presence of CHS and further improved by presence of Astemizole (Figure 9) resulting in an almost perfect symmetrical FSEC elution profile, indicative of channels that are highly qualified for initiating crystallization trials. The fact that CHS improved the FSEC profile of hERG channels so dramatically demonstrates the important role of CHS for maintaining membrane protein structure. The further improvement in monodispersity observed in presence of Astemizole is also very encouraging as it shows that all the solubilized protein has maintained its ability to bind this specific and high-affinity ligand. This strongly supports that the solubilization conditions we have developed maintain the functional tetrameric structure, in accordance with the elution of the solubilized hERG channel as a 620 kDa protein. A tetrameric structure is necessary for activity, and therefore an indication of functionality [38]. To our knowledge this is the first time hERG has been purified as a full length tetramer. Ni-affinity purification of FC-12/CHS solubilized hERG-TEV-GFP-His _8_ resulted in a very pure preparation as indicated by SDS-PAGE analysis as in-gel fluorescence and Coomassie staining showed a single protein band with the expected molecular weight of 156 kDa. No degradation products were observed indicating that the solubilization conditions did not expose the hERG-TEV-GFP-His _8_ channels to yeast proteases.

The ability to produce the hERG channel to a high membrane density is only relevant if the purified protein has preserved its biological activity. It was therefore encouraging that the capacity of the purified channel for Astemizole binding approached one binding site per four hERG-TEV-GFP-His _8_ protein chains as the binding capacity for this ligand has been shown to correlate well with patch clamp electrophysiological experiments [38]. The fact that the purified hERG channels bound Astemizole confirms the FSEC results. Compared to the hyperbolic binding curve for membrane embedded hERG-TEV-GFP-His _8_ (Figure 7) the binding curve to purified and detergent solubilized hERG followed a sigmoid binding curve (Figure 11). Sigmoid binding curves are characteristic of cooperative binding of ligands to multi subunit proteins. In this case though, the hERG sigmoid binding may be explained by the different conformations that the channel pore can attain (open, close, rotated and intermediate) [48] and indicate that the detergent solubilized channel pore is more flexible in detergent than in the biological membrane.

Removal of the GFP-His _8_ tag by TEV cleavage may be a prerequisite for obtaining crystal structures and we were indeed able to remove the GFP-His tag quantitatively by TEV digestion during removal of imidazole by dialysis (Figure 12).

## Conclusions

In conclusion, we describe a cost-effective, novel and efficient solubilization and purification protocol, which generates milligram amounts of correctly folded, full-length hERG protein after production in *S. cerevisiae*. The amount and in particular the quality of the produced hERG channels is to our knowledge unprecedented and presents a major breakthrough in the study of hERG, which may facilitate further functional studies and structure determination through crystallization. Availability of large amounts of prime quality hERG channels may also accelerate studies of transfer of full length channel protein into biomimetic membranes for sensor and separation applications [49]. Such a setup may be used for rapid screening to help exclude potential drugs with detrimental side effects at an early stage and restrict focus to candidates without such effects.

## Methods

### Yeast strains

Production of tagged protein constructs for purification was carried out in *S. cerevisiae* strain PAP1500 (*αura3-52 trp1::GAL10-GAL4 lys2-801 leu2Δ*1 his3 *Δ200 pep4::HIS3 prb1Δ1.6R can1 GAL*) [30] while complementation studies were performed in PAP7111 (*αura3-52 his3 Δ 200 HIS 4-15 trk1 Δ* trk2 *Δ::HIS3 PMA1::mcherry*). PAP7111 was constructed by transformation of CY162 [50] with a PCR fragment carrying the mCherry [51] coding region flanked by 35 nucleotides used for homologous recombination with the chromosomal *PMA1* locus. The wild type strain BY4741 (a *his3Δ1 leu2Δ0 met15Δ0 ura3Δ0*) [52] was used in the complementation studies, too.

### Recombinant plasmid construction

A 3480 bp long yeast codon optimized hERG sequence was purchased from Genscript, USA. To C-terminally tag hERG with a Tobacco Etch Virus cleavage site and a yEGFP-His _8_ sequence, we PCR amplified codon optimized hERG cDNA with primers hERGfw 5 ^′^-*ACACAAATACACACACTAAATTACCGGATCAATTC*TTTAAAACGA**ATGCCAGTTAGAAGAGG TC**-3 ^′^ and hERGrv 5 ^′^-*AAAT TGACTTTGAAAATACAAATTTTC***ACTACCTGGGTCACTACCG** - 3 ^′^ and yEGFP cDNA [32] with primers GFPfwTEV 5 ^′^-*AAAATTTGTATTTTCAAAGTCAATTT***TCTAAAGGTGAAGAATTATTCACT**-3 ^′^ and GFPHISdo 5 ^′^-*CTT CAATGCTATCATTTCCTTTGATATTGGATCAT*CTAATGGTGATG GTGATGGTGATGGTG**TTTGTACAATTCA**-3 ^′^.

The emphasized nucleotides were used for *in vivo* homologous recombination, the bold nucleotides are identical or inverse complimentary to the template, the text in between these two formattings in hERGfw is the Kozak sequence from the yeast *PMR1* gene and in the GFPHISdo it is the His-tag. The TEV site is marked in *italics*. All PCR reactions were performed with AccuPol DNA polymerase (Amplicon, Denmark). The hERG-TEV-GFP-His _8_ expression plasmid was generated by *in vivo* homologous recombination by transforming PAP1500 with hERG and GFP PCR products and *Sal*I, *Hin*dIII and *Bam*HI digested pEMBLyex4 [53] expression vector, using the transformation protocol described by Gietz and Schiestl [54]. The correct nucleotide sequence of the expression construct was verified by DNA sequencing at Eurofins MWG Operon, Germany.

### Yeast complementation assay

PAP7111 cells harboring the pEMBLYex4 plasmid, the hERG-TEV-GFP-His _8_ expression plasmid or the hERG-His _10_ expression plasmid were cultured in SD medium [32] supplemented with 100 mM KCl. The wild type strain BY4741 was cultured in SD medium supplemented with histidine, leucine, methionine and uracil. Cells were subsequently harvested, washed thoroughly with 18 m *Ω* H _2_O to remove residual KCl originating from the initial growth medium and inoculated in TES-TRIS buffered (pH 6.0) SD+SG medium at OD _450_=0.05 in 96 well micro plates (Nunc, clear plastic) at KCl concentrations of 0, 0.1, 1, 2, 5, 10, 15, 20, 25, 30, 35 or 100 mM, respectively. Growth was monitored 3 times a day for 5 days by measuring OD _450_.

### Recombinant hERG production

Yeast cells were cultured and induced to express hERG as described by Scharff-Poulsen, P and Pedersen, PA [32] In brief cells were inoculated in 5 ml synthetic minimal (SD) medium supplemented with leucine and incubated at 30°C O/N until saturation. The plasmid copy number in the yeast population was subsequently increased by growth in medium lacking leucine. This culture was used to inoculate 1 L of expression medium, which is SD medium with glucose (0.5% w/v), glycerol (3% v/v), alanine (20 mg/L), arginine (20 mg/L), aspartic acid (100 mg/L), cysteine (20 mg/L), glutamic acid (100 mg/L), histidine (20 mg/L), lysine (30 mg/L), methionine (20 mg/L), phenylalanine (50 mg/L), proline (20 mg/L), serine (375 mg/L), threonine (200 mg/L), tryptophane (20 mg/L), tyrosine (30 mg/L) and valine (150 mg/L) to an OD _450_ of 0.05. The culture was incubated at room temperature until the OD _450_ reached 1.0, transferred to 15°C and supplemented with induction medium (identical to the expression medium described above except that 20% galactose has substituted 0.5% glucose) to a final concentration of 2% galactose. The culture was incubated for at least 48 hours before harvesting.

### Live cell bioimaging

Localization of heterologously expressed GFP-tagged hERG was determined by visualizing GFP fluorescence in whole cells at 1000 × magnification, using a Nikon Eclipse E600 microscope coupled to an Optronics Magnafire model S99802 camera.

### Deglycosylation

80 *μ*g of crude membranes were incubated with 500 units of Endo-H (New Biolabs, USA) at 4°C in Lysis buffer over night alongside 80 *μ*g of crude membranes in lysisbuffer with no added Endo-H. Samples were separated in a 10% SDS-PAGE gel at 150 V for 2 hours, and visualized by in-gel fluorescence.

### Temperature optimization of hERG production

Yeast cells were grown at room temperature as described above in 1 L of expression medium. At OD _450_=1.0, half of the culture was transferred to 15°C and the other to 30°C. After thermo equilibration, hERG production was induced by adding 55 ml of induction medium. Samples were collected 12, 24, 48, 72 and 96 hours post induction. Crude membranes were isolated from cells harvested at each time point and analysed by in-gel fluorescence using a LAS 4000 (GE Healthcare, USA).

### Membrane preparation

Crude yeast membranes were prepared by disrupting cell pellets by glassbead vortexing [55]. Briefly, cell pellets from 1 L cultures were resuspended in 10 ml ice cold lysis buffer (25 mM imidazole, 1 mM EDTA, 1 mM EGTA, 10% glycerol (v/v) pH 7.5) with protease inhibitors (1 mM PMSF, 1 mM benzamidine, leupeptin (1 *μ*g/ml), pepstatin (1 *μ*g/ml), chymostatin (1 *μ*g/ml) and aprotinin (1 *μ*g/ml)). Samples were vortexed 4 × 1 minutes with at least 1 minute of cooling in between mixing. The liquid phase was collected, and beads were washed several times with lysis buffer generating samples of 50 ml total volume. Cell debris was pelleted by centrifugation for 10 minutes at 3,000 rpm and 4°C in an SS-34 rotor. Crude membranes were pelleted from the supernatant by ultra-centrifugation for 1.5 hour at 40,000 rpm and 4°C in a 70TI rotor. Crude membranes were resuspended in 3 ml lysis buffer with protease inhibitors (as above), homogenized in a Potter-Elvehjem homogenizer and stored at -80°C for further use.

### Protein and hERG-GFP quantification

Protein concentrations in crude membranes were determined by the BCA assay [56] according to the manufacturer ^′^s specifications (Sigma, USA) using BSA as a standard. The density of hERG-TEV-GFP-His _8_ in yeast membranes was determined from the GFP fluorescence emitted from 25 *μ*g of total membrane protein measured in 96 well white microplates (Nucleon Nunc) after adjustment of the volume to 200 *μ*l with buffer (20 mM phosphate pH 7.0, 200 mM NaCl, 10% glycerol, 10 mM Imidazole). Fluorescence was measured in a spectrofluorometer (Fluoroskan Ascent, Thermo Scientific) using buffer as a blank. Excitation was at 485 nm and emission at 520 nm. Fluorescence was converted to pmol hERG-GFP from a standard curve generated from purified GFP mixed with yeast membranes as previously established [31,32].

### Astemizole binding to crude membranes

Crude membranes were used to assess the capability of the recombinant hERG-GFP to bind the hERG ligand Astemizole, as described for HEK293 cells expressing hERG [38]. Aliquots of 200 *μ*g crude membrane protein in total volumes of 400 *μ*l incubation buffer (10 mM HEPES 130 mM NaCl, 60 mM KCl 0.8 mM MgC l_2_ 10 mM glucose 1 mM EGTA pH 7.4) supplemented with protease inhibitors (1 mM PMSF, 1 mM Benzamidine, 1 *μ*g/ml Leupeptin, Chymostatin, Pepstatin and Aprotinin) were mixed with [ ^3^H]-Astemizole concentrations ranging from 0.5 - 20 nM. Unspecific binding was determined in the presence of 10 *μ*M non-radiolabeled Astemizole. Binding was done at 15°C for 2 hours with slow speed shaking, and samples kept on ice here on after. Protein-ligand complexes were separated from free ligand by filtration of 200 *μ*l sample through Whatman GF/B glass fiber filters presoaked in 0.3% polyethyleneimine and washed once in 1 ml ice cold wash buffer (25 mM Tris-HCl, 130 mM NaCl, 5 mM KCl, 0.8 mM MgC l_2_, 0.05 mM CaCl _2_, pH 7.4) with protease inhibitors. Subsequently filters were washed 6 times with 1 ml ice cold wash buffer using vacuum filtration, and bound ligand was detected using a Perkin Elmer Tri-Carb 2910 TR liquid scintillation counter. 50 *μ*l of unfiltered sample was used for determination of total CPM counts. A subsequent BCA protein determination assay was done on all samples to allow for corrections due to any protein loss during the binding assay. After calculating total, unspecific and specific binding the resulting graph was analyzed using the Sigmaplot non-linear regression tool, ligand binding; one-site saturation (f = Bmax*abs(x)/(Kd + abs(x)) to estimate binding affinity and capacity.

### Astemizole binding to purified protein

0.1 *μ*g of affinity purified hERG-TEV-GFP-His _8_ protein was used to estimate binding affinity and binding capacity. Purified hERG protein was incubated with increasing ammounts of [ ^3^H]-Astemizole, filtered and [ ^3^H]-Astemizole binding determined by scintillation counting. Unspecific binding was determined in presence of 10 *μ*M non-radioactive labelled Astemizole. All solutions contained 1.5 mg/ml FC-12 and 0.5 mg/ml CHS. Experimental data were analysed in Sigmaplot using a sigmoid 3 parameter curve-fit (f=a(1+exp(-(x-x _0_)/b).

### Detergent screening

Crude membranes were incubated in buffer B (25 mM Tris-HCl, 10 mM Imidazole, 0.5 M NaCl, 10% glycerol, pH 7.6) supplemented with protease inhibitors (1 mM PMSF, 1 mM Benzamidine and 1 *μ*g/ml Leupeptin, Chymostatin, Pepstatin and Aprotinin respectively) at protein:detergent:CHS ratios (w/w) of 1:2:0.7; 1:3:1 or 1:4:1.4 The screen included detergents FC-12, n-dodecylphosphocholine; LDAO, Lauryldimethylamine N-oxide; Cymal-5, 5-cyclohexyl-1-pentyl- *β*-D-maltoside; DDM, n-dodecyl- *β*-D-maltopyranoside; DM, n-decyl- *β*-Dmaltopyranoside; C _12_*E*_8_, Octaethylene glycol monododecyl ether; CHAPS, 3-[(3chol-amidopropyl)-dimethylammonio]1-propane sulfonate/ N,N-dimethyl-3-sulfo-N-[3-[[3a,5b,7a,12a)-3,7,12- tri - hydroxy-24-oxocholan-24-yl]amino]propyl]-1-propana- miniumhydroxide and Octyl glucoside. All detergents were of Anagrade quality and purchased from Affymetrix, UK. Solubilization was performed at slow rotation at 4°C for 1 hour. Solubilized hERG-GFP channel protein was separated from un-solubilized cell debris by ultra-centrifugation at 70,000 rpm for 30 minutes at 4°C in a Beckman Optima™TLX ultracentrifuge fitted with an S.N. 96U 826 rotor. Fluorescence was detected in microplates in a spectrofluorometer (Fluoroskan Ascent, Thermo Scientific) using buffer as a blank. Excitation was at 485 nm and emission at 520 nm. Solubilization efficiency was etimated as fluorescence in the supernatant divided by fluorescence in the crude membranes used for solubilization.

### FSEC

Solubilized crude membranes were analyzed by fluorescence size exclusion chromatography (FSEC) on a Superose 6 10/300 column attached to an $\ddot {A}$KTA Purifier (GE Healthcare, USA), using FSEC buffer (20 mM TRIS-HCl, 0.15 M NaCl, 0.03% DDM). 1 *μ*M Astemizole was added to the buffer in experiments involving Astemizole. The effluent from the Superose 6 10/300 column was coupled to a fluorescence detector (Shimadzu Prominence RF-20A), to measure fluorescence and visualize the elution profile of the GFP tagged hERG channel. To estimate the molecular weight of the solubilized hERG-TEV-GFP-His _8_ protein, we used the HMW calibration kit from GE Healthcare dissolved at 20 mg/ml in FSEC buffer. The molecular masses were: Ovalbumin 43 kDa; Conalbumin 75 kDa; Aldolase 158 kDa; Ferritin 440 kDa; Thyroglobulin 669 kDA; Blue Dextran 2000 kDa. The elution volume for Blue Dextran defined the void volume.

### Ni-NTA affinity purification

For purification, the hERG-GFP protein was solubilized in buffer B at a protein:FC-12:CHS ratio of 1:3:1 (w/w/w) at slow rotation at 4°C for 1 hour. Non-solubilized material was pelleted at 70,000 rpm in the Beckmann Optima TL200 ultracentrifuge for 30 minutes at 4°C. Solubilized membranes were diluted in buffer B with protease inhibitors to a detergent concentration of 0.75 mg/ml corresponding to 1.5 times CMC for Fos-choline-12 and a CHS concentration of 0.26 mg/ml, incubated over night with 1 ml of Ni-NTA Agarose (Qiagen, Germany) at 4°C with slow magnetic stirring. The Agarose slurry was subsequently loaded onto a 2 ml CellThru disposable column (Clontech, USA). After collection of the run through, the column was washed with Buffer B containing 10 mM, 30 mM, 100 mM, 250 mM or 500 mM imidazole. All buffers contained 0.75 mg/ml FC-12 and 0.26 mg/ml CHS Fluorescence in each fraction was quantified using a spectrofluorometer (Fluoroskan Ascent, Thermo Scientific) using buffer as a blank. Excitation was at 485 nm and emission at 520 nm.

### TEV cleavage

Purified hERG-GFP-His _8_ fusion protein were digested O/N in snakeskin dialysis bags (Thermo Scientific, USA) with dialysis buffer (20 mM phosphate pH 7.0 200 mM NaCl 0.075% (w/v) FC-12 0.026% (w/v) CHS) and TEV protease [32] at room temperature with a protein to TEV ratio of 1:10 (w/w). Digestion efficiency was estimated by in-gel fluorescence followed by Coomassie staining.

## Abbreviations

hERG: human *Ether-à-go-go* related gene
GFP: Green fluorescent protein
FSEC: Fluorescence size exclusion chromatography
TEV: Tobacco etch virus
TM: Transmembrane
FC-12: Fos-Choline 12
CHS: Cholesteryl-hemisuccinate
CPM: Counts per minute
